# Orthology between genomes of *Brachypodium*, wheat and rice

**DOI:** 10.1186/1756-0500-2-93

**Published:** 2009-05-27

**Authors:** Sachin Kumar, Amita Mohan, Harindra S Balyan, Pushpendra K Gupta

**Affiliations:** 1 Molecular Biology Laboratory, Department of Genetics and Plant Breeding Ch. Charan Singh University, Meerut-250 004, India

## Abstract

**Background:**

In the past, rice genome served as a good model for studies involving comparative genomics of grass species. More recently, however, *Brachypodium distachyon *genome has emerged as a better model system for genomes of temperate cereals including wheat. During the present study, *Brachypodium *EST contigs were utilized to resolve orthologous relationships among the genomes of *Brachypodium*, wheat and rice.

**Findings:**

Comparative sequence analysis of 3,818 *Brachypodium *EST (bEST) contigs and 3,792 physically mapped wheat EST (wEST) contigs revealed that as many as 449 bEST contigs were orthologous to 1,154 wEST loci that were bin-mapped on all the 21 wheat chromosomes. Similarly 743 bEST contigs were orthologous to specific rice genome sequences distributed on all the 12 rice chromosomes. As many as 183 bEST contigs were orthologous to both wheat and rice genome sequences, which harbored as many as 17 SSRs conserved across the three species. Primers developed for 12 of these 17 conserved SSRs were used for a wet-lab experiment, which resolved relatively high level of conservation among the genomes of *Brachypodium*, wheat and rice.

**Conclusion:**

The present study confirmed that *Brachypodium *is a better model than rice for analysis of the genomes of temperate cereals like wheat and barley. The whole genome sequence of *Brachypodium*, which should become available in the near future, will further facilitate greatly the studies involving comparative genomics of cereals.

## Background

Cereals constitute the most important group of cultivated plants, and are known to have diverged from a common paleopolyploid ancestor ~45–47 million years ago (Mya) [1]. Despite this, a remarkable overall structural and functional similarity exists among different cereal genomes [2,3], although the size of these genomes differs greatly, ranging from 430 Mb in rice (*Oryza sativa*) to 16,000 Mb in hexaploid wheat (*Triticum aestivum*). Due to its small size and availability of whole genome sequence, rice has been used as a model system for a variety of experimental studies including map-based cloning [4]. However, recent studies resolved further the dynamic changes in rice genome sequences, thus questioning the utility of rice as a model crop [5], and necessitating the need for search of a more efficient model system.

*Brachypodium distachyon*, a small temperate grass (sub-family Pooideae) has recently emerged as a better model system for the study of temperate grasses. This is particularly, due to several of its desirable biological features and its phylogenetic position [6,7]. It is postulated that relative to rice genome, *Brachypodium *genome will exhibit a much higher level of colinearity and synteny to the genomes of temperate cereal crops. In the present study, the available *Brachypodium *EST contigs (bEST contigs) and supercontigs were utilized to explore further the utility of the *Brachypodium *genome as a model for carrying out comparative genomics studies in cereals in general, and for wheat genomics in particular. The relationship of *Brachypodium *genome with wheat and rice genomes has been examined for this purpose, and improved criteria of sequence similarity search were used for more accurate estimation of similarity [8].

## Results

In the present study, EST sequences from *Brachypodium *were utilized to find out the degree of similarity of *Brachypodium *genome with EST/genomic sequences of wheat and rice. The orthologous wheat sequences thus identified were also utilized to study the relationship of wheat genome sequences with *Brachypodium *supercontigs. We have also taken note of the comparisons of chloroplast genomes among eight grass species, which were included in the report on *Brachypodium *chloroplast genome sequence that was recently worked out [9].

### Orthology between *Brachypodium *and wheat

As many as 3,818 *B. distachyon *EST contigs were blasted (BLASTN) against the available wheat EST contigs (containing bin-mapped wESTs) to identify matching wESTs. The analysis revealed that as many as 449 bEST contigs had orthologs in wheat genome.

### Analysis of mapped wEST contigs that matched bEST contigs

**Figure 1:**
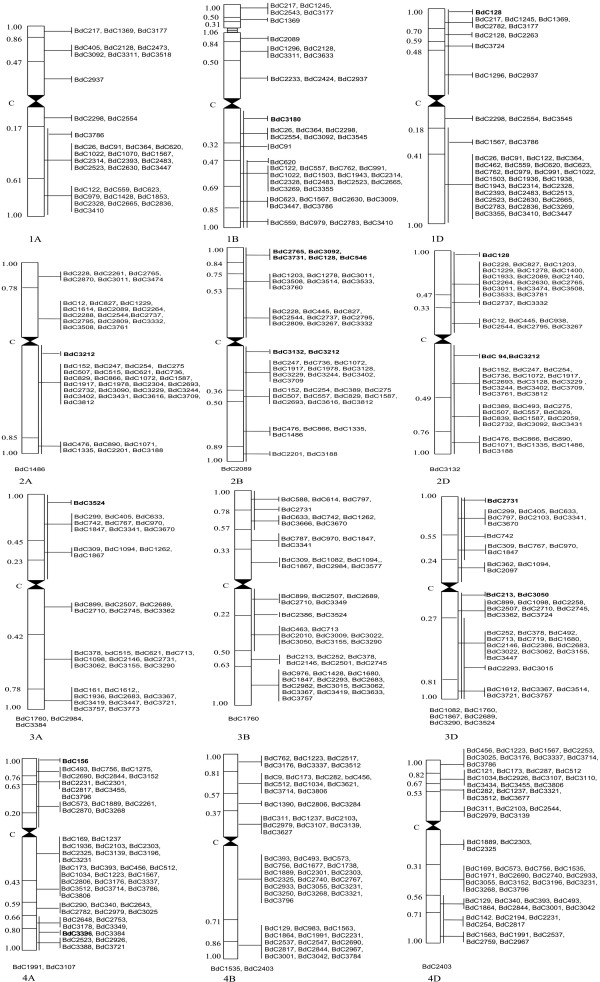
**Distribution of orthologous bEST contigs (BdC) on wheat chromosomes belonging to homoeologous groups 1 to 4 (12 chromosomes)**. bEST contigs are shown on the right and arm fraction lengths are given on the left. Vertical lines on the right, covering an arm, means that the corresponding bEST contig (shown in bold) could not be assigned to a specific bin and was assigned to the arm; vertical lines covering more than one bins means that corresponding wEST was earlier mapped to a 'combined bin', rather than to an individual bin. The bEST contigs, which could not be assigned to bins and were assigned to individual chromosomes (with no information about arm), are listed at the bottom of each such individual chromosome.

**Figure 2:**
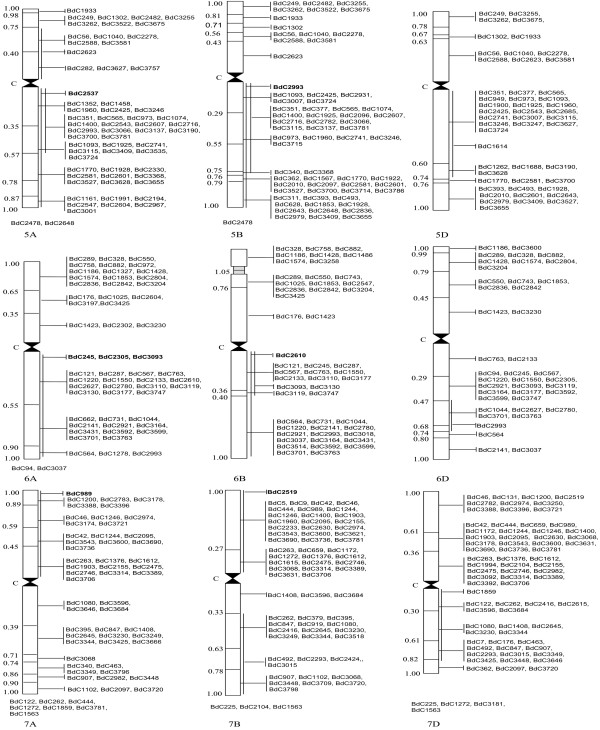
**Distribution of orthologous bEST contigs (BdC) on wheat chromosomes belonging to homoeologous groups 5 to 7 (9 chromosomes)**. bEST contigs are shown on the right and arm fraction lengths are given on the left. Vertical lines on the right, covering an arm, means that the corresponding bEST contig (shown in bold) could not be assigned to a specific bin and was assigned to the arm; vertical lines covering more than one bins means that corresponding wEST was earlier mapped to a 'combined bin', rather than to an individual bin. The bEST contigs, which could not be assigned to bins and were assigned to individual chromosomes (with no information about arm), are listed at the bottom of each such individual chromosome.

The above 449 bEST contigs were homologous with a corresponding number of wESTs carrying 1,154 bin-mapped loci or regions giving an average of 2.57 loci per wEST contig (Figures 1, 2). The distribution of ortholoci on the three wheat sub-genomes (A, B and D) and among the seven homoeologous groups of chromosomes (Table 1) was non-random (P << 0.05), when the known chromosome lengths and their DNA contents were used as the basis [10]. The distribution of ortholoci on long and short arms of the chromosomes (excluding 37 loci, which could not be assigned to individual arms) was also non-random (P < 0.05). This non-random distribution of ortholoci is, however, based on limited data.

**Table 1:** Distribution of the orthologous loci according to their assignment to wheat chromosomes arranged in two-way classification

	**Sub-genome**			
		
**Homoeologous group**	**A**	**B**	**D**	**Total**
1	36	46	46	**128**
2	53	51	62	**166**
3	43	54	52	**149**
4	59	61	66	**186**
5	62	65	49	**176**
6	57	51	41	**149**
7	59	66	75	**200**

**Total**	**369**	**394**	**391**	**1,154**

Of the above 449 matched wEST contigs (orthologous to bEST contigs), 77 (17.2%) represented unique loci, and the remaining 372 (82.8%) detected multiple loci with 283 (76.1%) having multiple loci on homoeologous chromosomes and 89 (23.9%) having multiple loci on non-homoeologous chromosomes.

Of the 1,154 orthologous loci with known positions on wheat chromosomes, 1,094 (94.8%) loci were known to have earlier been assigned to 159 chromosome bins defined by deletion break points. The remaining 60 (5.2%) loci could be assigned only to individual chromosomes or their arms. A maximum of 386 loci (35.3%) were mapped in the proximal regions (60% of the arm length from centromere; C-0.60) followed by 331 loci (30.3%) mapped to the distal regions (40% terminal arm length; 0.60–1.00). The remaining 377 loci (34.4%) were mapped to the interstitial bins having proximal and distal regions.

The above 449 mapped wheat orthologs were also used for homology search among *Brachypodium *supercontigs. The wheat EST contigs located on homoeologous group 4 chromosomes had maximum homology (54.5% of mapped contigs) with the *Brachypodium *super_1 contig. In contrast, *Brachypodium *super_0 to 2 contigs had homology with wEST contigs dispersed on all the seven homoeologous groups, although no redundancy for wheat homologues was observed within the above supercontigs (Table 2).

**Table 2:** Homology between *Brachypodium *supercontigs and homoeologous groups of wheat

**Wheat homoeologous group**	**bEST mapped contig**	Number of *Brachypodium *supercontigs showing orthology*	**Supercontig showing maximum homology**
1	55	11 (0–4,7–10,12,14)	2 (32.7%)
2	69	11 (0–6,9,10,14,15)	0 (44.9%)
3	63	11 (0–2,4–6,8–10,12,13)	4 (33.3%)
4	77	10 (0–3,6–8,11,15,189)	1 (54.5%)
5	68	11 (0–3,5–8,12,15,525)	0 (27.9%)
6	54	11 (0–9,11)	5 (42.5%)
7	63	15 (0–9,11,13,14,538)	0 (19.0%)

*Designated numbers for supercontigs are given in a parenthesis in each row

### Orthology between bEST contigs and rice genome sequences

**Figure 3:**
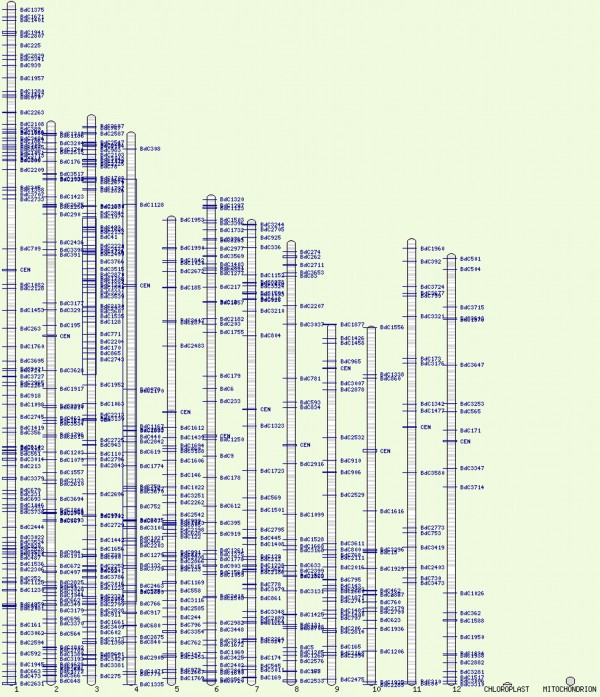
**Distribution of orthologous bEST contigs (BdC; shown on the right side) on 12 rice chromosomes**.

The BLASTN results of 3,818 bEST contigs against the rice genome sequences identified as many as 743 matching bEST contigs (see methods), which had homologues distributed on all the 12 chromosomes of rice. On the basis of relative length (Mb) of chromosomes and their arms [11], the ortholoci on 12 rice chromosomes/arms were non-randomly distributed (P <<< 0.05) (Table 3; Figure 3).

**Table 3:** Distribution of the orthologous loci on individual rice chromosome

**Rice Chromosome**	1	2	3	4	5	6	7	8	9	10	11	12	**Total**
**Number of loci**	122	95	133	52	64	61	52	43	41	23	27	30	**743**

### Conserved orthologous sequences among Brachypodium, wheat and rice

**Figure 4:**
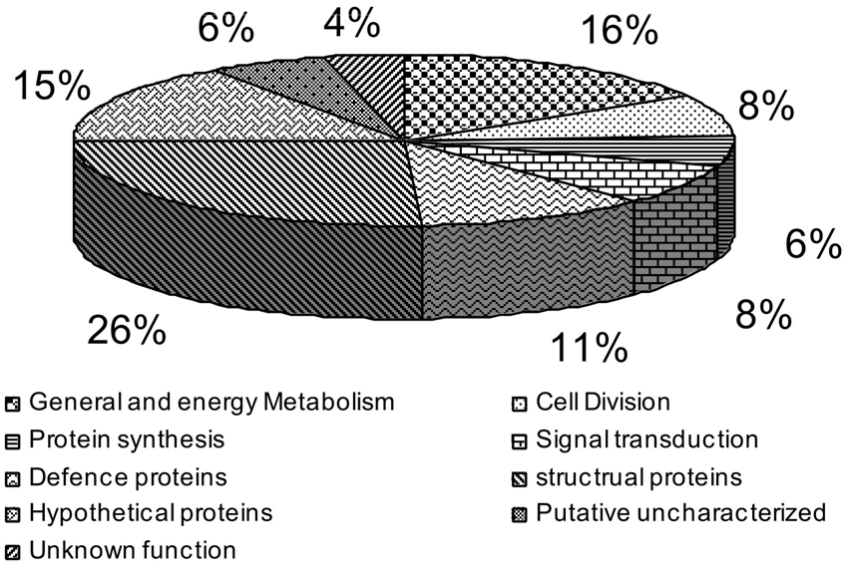
**A pie-chart showing relative frequencies (%) among 183 bEST orthologous sequences based on different biological functions and molecular activities**.

In the present study, 183 orthologous sequences were conserved among all the three species (*Brachypodium*, wheat and rice). As many as 126 of the 183 orthologous sequences also confirmed known homology between wheat-rice chromosomes. Functional annotation of these 183 orthologous sequences suggested that a majority (137; 74.8%) of these bEST contigs matched with proteins of known functions (see Additional file 1; Figure 4).

### Conservation of SSRs among the three genomes

The 183 bEST contig sequences shared by three species (*Brachypodium*, wheat and rice) were also used for mining SSRs. A total of 100 (54.6%) bEST contigs contained 137 SSRs. As many as 45 of these SSRs showed conservation in wheat and 23 of these SSRs showed conservation in rice. As many as 17 SSRs were conserved across all the three species.

### Transferability of conserved orthologous SSRs

**Figure 5:**
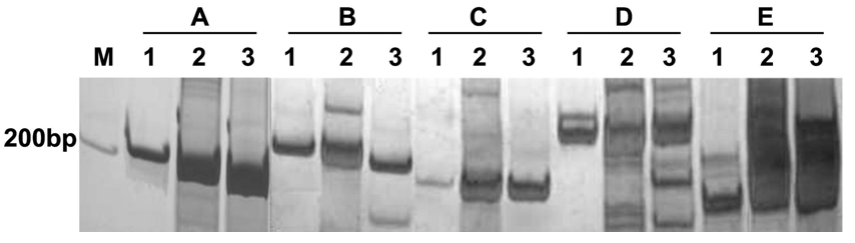
**A representative pattern of *Brachypodium *SSR marker PCR products showing conservation and cross-transferability in the genomes of wheat and rice**. Lane M, 100 bp DNA ladder; lane 1, *Brachypodium *DNA (Bd 21); lane 2, wheat DNA (Chinese Spring); lane 3, rice DNA (IR-1). The primers (L/R) used were (A) BDEST01P1_Contig9; (B) BDEST01P1_Contig1223; (C) BDEST01P1_Contig2416; (D) BDEST01P1_Contig3247; (E) BDEST01P1_Contig3747 (Table 4).

In order to validate experimentally the conservation of *Brachypodium *SSRs among the genomes of wheat and rice, primer pairs for SSRs belonging to 12 orthologs were synthesized and used for PCR amplification of the SSRs (Table 4). All the 12 primer pairs gave amplification products in wheat and rice (Figure 5).

**Table 4:** List of primers for the 12 conserved SSRs used for wet-lab experiment

***Brachypodium *contig Id**.	**Motif**	**Primer sequence 5'-3'**	**Tm (°C)**	**Product size (bp)**	**Gene class**
BDEST01P1_Contig9	(gct)5	L GCCTATGTTTCCGCAGAGAG R CCAGGCAAGAAGTTCCTGAG	59.98	203	Formate dehydrogenase
BDEST01P1_Contig1223	(cca)4	L AGCCAACTCTTGCAGCAAAT R TGTTGCTCCCTCCTTTTGAT	59.67	207	Endo-1,4-beta-glucanase Cel1
BDEST01P1_Contig1335	(tca)4	L GACGAGAGGTTGTGTTGGTG R ACAGGACACCGTCAGAGGAA	60.71	239	Putative uncharac-terized protein
BDEST01P1_Contig1574	(cgc)6	L CAAAACCCTAGCTGCCCTTC R TGCCAGTGCTTCTTGAAATG	59.99	123	Putative 60S ribos-omal protein L13E
BDEST01P1_Contig2089	(ggc)4	L GCTCTTCTCGCCCCTCTACT R CTCCATCTGGAAATCGCAGT	60.22	200	Hypothetical protein
BDEST01P1_Contig2416	(tcaaga)2	L CCGCACCTCAAGGACTACA R TCGGAGGAGATCTTGGTGAG	60.34	194	Succinate dehydrogenase
BDEST01P1_Contig2648	(ggt)4	L AAACCACTTGCCAAAACACC R GCTGCGGTTCTCCATGAC	60.37	248	Putative uncharac-terized protein
BDEST01P1_Contig3139	(tcg)4	L AGTCACCAAGGTCGTCAAGG R CCTTCGCTGCTCCATAGTCT	59.6	224	Putative ribosomal protein
BDEST01P1_Contig3247	(tggtgc)2	L AGTTGGAATGAGGGCATCAG R TTCAAGGCTCTCGAGTAGGG	59.57	214	Putative uncharac-terized protein
BDEST01P1_Contig3321	(gctcgc)2/N36/(ggc)4	L CACTTCGAGTTTCCCGTCAT R TTTTGCAGTGTCCACACCAT	60.01	244	Protein disulfide isomerase 2 precursor
BDEST01P1_Contig3721	(gct)4	L GGACTACTTTGGGGCTCACA R GGATTCATAACTGGCAACCA	59.44	180	Cytosolic 6-phosphogluconate dehydrogenase
BDEST01P1_Contig3747	(tcgcca)2	L AGGTCAACTCGGTCAACGAC R AGGTCAGCCCGTTGTTGTAG	60.17	192	Phenylalanine ammonia-lyase

## Discussion

Comparative genomics among grasses initially focused on the analysis of colinearity (gene order) and synteny (gene content) among DNA markers mapped on individual chromosome at a low resolution (10 cM). This led to the identification of 30 rice-independent linkage blocks involved in the constitution of all cereal genomes and allowed identification of a number of rearrangements within individual genomes [12]. However, due to the availability of whole genome sequence of rice, and substantial partial sequences from other cereal genomes, emphasis shifted to a comparison of nucleic acid sequences. In particular, sequences of ~7000 bin-mapped wESTs were aligned with rice genome sequences [13], allowing improved resolution and discovery of many more rearrangements.

Although rice worked well as a model for all grasses including wheat, and generated useful information, *Brachypodium*, belonging to subfamily Pooideae (wheat also belongs to Pooideae), is proposed as a better model than rice (subfamily Ehrhartoideae). Recent studies have suggested that relative to rice, *Barchypodium *is more closely related to wheat and barley and the colinearity between *Barchypodium *and wheat is better than that between wheat and rice [14,15]. Chloroplast sequence-based phylogenetic analysis in eight grass species also suggested that *Brachypodium *is closer to the tribe Triticeae [9]. The possible estimated time of divergence between *Brachypodium *and Triticeae is also shorter (35 Mya) than that of divergence between wheat and rice (50 Mya) [16] thus supporting the view that *Brachypodium *is more closely related with the members of Triticeae.

During the present study, orthologous relationship among bEST contigs, wEST contigs and rice genome sequences was studied using improved criteria of sequence comparison. Observation of higher number of bEST contigs showing orthology with rice genome was mainly attributed to the fact that only a small fraction of wheat genome (0.02%) and almost complete rice genome (95%) were used for sequence comparison with the available *Brachypodium *EST contigs. If we take into account the proportion of the genome used for comparison, it may be concluded that wheat has higher level of orthology with *Brachypodium *than with rice.

The mapped loci in different deletion bins of a particular chromosome of wheat matched with same or different supercontigs of *Brachypodium*. For instance, wheat group 4 chromosomes are highly syntenic to *Brachypodium *super_1 contig (54.5%) than to other supercontigs, although super_1 contig showed homology with other homoeologous groups also. The mapping information of these *Brachypodium *supercontigs on individual *Brachypodium *chromosomes will be useful for developing markers specific to the targeted regions of wheat chromosomes.

It was also observed that although D sub-genome of wheat is smaller in size, the orthologous loci mapped on this sub-genome are no fewer than those mapped on sub-genome B, suggesting closer relationship between *Brachypodium *and *Aegilops tauschii*, the donor of the D sub-genome of hexaploid wheat.

The relative abundance of orthologous loci on proximal regions of chromosome arms in wheat is in agreement with the earlier studies in wheat and rice [17]. It seems that higher degree of sequence conservation coincides with the low recombination proximal regions, which is understandable, since high recombination in terminal regions will cause reshuffling of genes during evolution [18].

## Conclusion

The results of the present study indicate that the availability of whole genome sequence of *Brachypodium *will be of enormous relevance for comparative genomics, gene annotation and evolutionary, structural and functional genomic studies of large genomes of the Triticeae.

## Methods

### *Brachypodium*, wheat, rice sequence databases

A total of 3,818 *Brachypodium *EST (bEST) contigs, and a set of 1,015 supercontigs representing 4× coverage of *Brachypodium *genome, were available in public domain [19,20]. As many as 3,792 wheat EST (wEST) contigs containing bin-mapped wESTs were available at GrainGenes 2.0 [21] and rice genomic sequences were available at Gramene [22].

### Sequence comparisons

In order to find orthology among *Brachypodium*, wheat and rice genomes, bEST contigs were blasted against wEST contigs and rice genomic sequences. The pairwise sequence alignment in BLASTN search was improved by using three new parameters [8]. The first parameter, aligned length (AL), corresponds to the sum of the lengths of all the high-scoring segment pairs (HSPs) in a single hit. Second parameter, cumulative identity percentage (CIP) was obtained from the formula, CIP = [Σ Id of HSPs/AL] × 100 and the third parameter, cumulative alignment length percentage (CALP) was calculated as follows: CALP = [AL/QL] × 100, where, QL is the length of query sequence. Last two parameters (CIP and CALP) allow estimation of highest similarity between sequences over the entire length of query sequence. These parameters were applied to all the BLASTN results and values of 60% CIP and 70% CALP were used for identification of orthologs of *Brachypodium *genomic sequences in wheat (through ESTs) and rice genomes.

### Mapping of wheat and rice orthologs

The physical positions of wEST orthologs identified through sequence comparisons were localized to specific bins of wheat chromosomes based on the information about mapped wEST sequences [23]. The rice genomic sequences, which were orthologous to bEST contigs, were also known and were physically localized to specific sites on 12 different rice chromosomes with the help of KaryoView program [24]. The χ^2 ^test for goodness-of-fit was used for testing the random distribution of ortholoci in wheat genome at the level of the three sub-genomes, the seven homoeologous groups, the 21 chromosomes and the 42 chromosome arms. The same was done for the 12 chromosomes of rice.

### Assignment of putative function to orthologs

The orthologous sequences belonging to the three genomes (*Brachypodium*, wheat and rice) were subjected to BLASTX analysis against non-redundant protein database [25] for assigning putative functions at a cut-off E value of 10^-30^.

### Identification of SSRs in orthologs

The orthologous sequences available in all the three genomes were mined for simple sequence repeats (SSRs) using SSRIT program [26]. The SSRs with a repeat motif of 2–6 nucleotides and a length of ≥ 12 bp were included in the analysis. Primers were designed for the 12 conserved SSRs using PRIMER3 [27].

### Wet-lab analysis

Primers for 12 conserved *Brachypodium *SSRs were synthesized from Invitrogen, USA. PCR was performed separately using the genomic DNA of *Brachypodium*, wheat and rice in a final volume of 20 μl in an Applied Biosystems 'Veriti Thermal Cycler'. After electrophoresis, polyacrylamide gels were silver stained following Tegelstrom [28].

## Competing interests

The authors declare that they have no competing interests.

## Authors' contributions

SK and AM participated in the design of the study, performed analysis and drafted the manuscript. HSB and PKG participated in the design and supervision of the study and preparation of the final manuscript. All authors have read and approved the final manuscript.

## Supplementary Material

Additional file 1 **Conserved bEST contigs, their locations on wheat and rice chromosomes and their annotated functions.** Click here for file
