# Supplementary material for: Orthology between genomes of Brachypodium, wheat and rice
Source: BMC Res Notes. 2009 May 27;2:93. doi: 10.1186/1756-0500-2-93 (PMC2695472; doi:10.1186/1756-0500-2-93)
Supplement: Additional file 1 — Conserved bEST contigs, their locations on wheat and rice chromosomes and their annotated functions. [file 1756-0500-2-93-S1.doc]

**Additional file 1:** Conserved bEST contigs, their locations on wheat and rice chromosomes and their annotated functions

| ***Brachypodium* contig** | **Homoeologous group**  **of wheat chromosomes** | **Rice chromosome** | **Annotated function** |
| --- | --- | --- | --- |
| BDEST01P1_Contig5 | 7 | 8 | Signal recognition particle receptor beta subunit-like protein |
| BDEST01P1_Contig9 | 4,7 | 6 | Formate dehydrogenase |
| BDEST01P1_Contig122 | 1,7 | 5 | Hypothetical protein |
| BDEST01P1_Contig128 | 1,2 | 3 | Putative nucleolar protein |
| BDEST01P1_Contig129 | 4 | 7 | Beta-amylase (1 and 4-alpha-D-glucan maltohydrolase) |
| BDEST01P1_Contig131 | 7 | 8 | Caffeoyl-CoA O-methyltransferase |
| BDEST01P1_Contig142 | 4 | 2 | Catalase isozyme A (CAT-A) |
| BDEST01P1_Contig152 | 2,6,7 | 6 | Putative polyubiquitin |
| BDEST01P1_Contig161 | 3 |  | Fructose-bisphosphate aldolase cytoplasmic isozyme |
| BDEST01P1_Contig169 | 4 | 7 | Hypothetical protein |
| BDEST01P1_Contig173 | 4 | 11 | Chlorophyll a/b-binding protein CP26 precursor |
| BDEST01P1_Contig176 | 6,7 | 2 | Eukaryotic initiation factor 4A-1 |
| BDEST01P1_Contig213 | 3 | 1 | Hypothetical protein |
| BDEST01P1_Contig217 | 1 | 6 | 12-oxo-phytodienoic acid reductase |
| BDEST01P1_Contig225 | 7 | 1 | Histone H2B.3 |
| BDEST01P1_Contig228 | 2 | 7 | Ribosomal protein s6 RPS6-2 |
| BDEST01P1_Contig245 | 6 | 4 | PIP aquaporin isoform |
| BDEST01P1_Contig252 | 3 | 1 | Putative Ras-related GTP-binding protein RAB11C |
| BDEST01P1_Contig254 | 2,7 | 6 | Putative polyubiquitin |
| BDEST01P1_Contig262 | 7 | 8 | Cytoplasmatic ribosomal protein S13 |
| BDEST01P1_Contig263 | 7 | 1 | Hypothetical protein |
| BDEST01P1_Contig275 | 2 | 3 | Putative proteasome regulatory non-ATPase subunit |
| BDEST01P1_Contig289 | 6 | 2 | Calcium-dependent protein kinase |
| BDEST01P1_Contig290 | 4 | 2 | 26S proteasome RPT6a subunit |
| BDEST01P1_Contig309 | 3 | 1 | Hypothetical protein |
| BDEST01P1_Contig329 | 7 | 1 | Histone H2B.2 |
| BDEST01P1_Contig340 | 3,4,7 | 1 | Beta-tubulin |
| BDEST01P1_Contig351 | 2,5 | 12 | Hypothetical protein |
| BDEST01P1_Contig362 | 3,5,7 | 12 | Hypothetical protein |
| BDEST01P1_Contig377 | 5 | 9 | Protein phosphatase 2A regulatory subunit A |
| BDEST01P1_Contig378 | 3 | 1 | Unnamed protein product |
| BDEST01P1_Contig379 | 7 | 6 | Putative T complex protein |
| BDEST01P1_Contig389 | 2 | 1 | Pathogenesis related protein |
| BDEST01P1_Contig393 | 4,5 | 3 | Hypothetical protein |
| BDEST01P1_Contig395 | 7 | 6 | Ras-related protein RIC2 |
| BDEST01P1_Contig444 | 7 | 8 | Heat shock protein XF20-1 |
| BDEST01P1_Contig445 | 2 | 7 | 60S ribosomal protein L44 |
| BDEST01P1_Contig456 | 4 | 12 | Putative UDP-glucose dehydrogenase |
| BDEST01P1_Contig462 | 1 | 10 | 60S ribosomal protein L21 |
| BDEST01P1_Contig463 | 3,7 | 2 | Putative elongation factor 2 |
| BDEST01P1_Contig493 | 2,4,5 | 3 | Glutamate decarboxylase |
| BDEST01P1_Contig515 | 2,3 | 5 | Unknow protein |
| BDEST01P1_Contig550 | 6 | 1 | Unknown |
| BDEST01P1_Contig564 | 6 | 2 | Putative 1-aminocyclopropane-1-carboxylate deaminase |
| BDEST01P1_Contig565 | 2,5 | 12 | Ribulose-1,5-bisphosphate carboxylase/oxygenase small subunit |
| BDEST01P1_Contig567 | 6 | 2 | Putative nitrilase 2 |
| BDEST01P1_Contig623 | 1 | 10 | Hypothetical protein |
| BDEST01P1_Contig628 | 5 | 5 | Hypothetical protein |
| BDEST01P1_Contig633 | 3 | 8 | Hypothetical protein |
| BDEST01P1_Contig662 | 6 | 2 | Hypothetical protein |
| BDEST01P1_Contig736 | 2 | 2 | Ribosomal subunit 8E protein |
| BDEST01P1_Contig762 | 1,4 | 5 | Phosphoethanolamine methyltransferase |
| BDEST01P1_Contig763 | 6 | 2 | Putative 60S ribosomal protein L6 (RPL6C) |
| BDEST01P1_Contig767 | 3 | 3 | YOR3513c, putative, expressed |
| BDEST01P1_Contig797 | 3 | 9 | 60S ribosomal protein L9 |
| BDEST01P1_Contig827 | 2 | 7 | Hypothetical protein |
| BDEST01P1_Contig890 | 2 | 4 | Pyruvate kinase |
| BDEST01P1_Contig949 | 5 | 7 | Hypothetical protein |
| BDEST01P1_Contig970 | 3 | 1 | Unknown protein |
| BDEST01P1_Contig979 | 1 | 5 | Mitogen-activated protein kinase 9 (MAP kinase 9) |
| BDEST01P1_Contig983 | 4 | 3 | 60S ribosomal protein L21 |
| BDEST01P1_Contig991 | 1 | 5 | Putative actin 1 |
| BDEST01P1_Contig1022 | 1 | 5 | UDP-glucuronic acid decarboxylase |
| BDEST01P1_Contig1034 | 4 | 11 | Precursor of CP29, core chlorophyll a/b binding (CAB) protein of photosystem II |
| BDEST01P1_Contig1044 | 6 | 2 | Hypothetical protein |
| BDEST01P1_Contig1071 | 2 | 4 | Pyruvate kinase |
| BDEST01P1_Contig1082 | 3 | 1 | ATP-dependent Clp protease proteolytic subunit |
| BDEST01P1_Contig1093 | 1,3,4,5 | 3 | Actin-3 |
| BDEST01P1_Contig1098 | 3 | 1 | Dihydroflavonol 4-reductase |
| BDEST01P1_Contig1186 | 6 | 2 | Putative 15.9 kDa subunit of RNA polymerase II |
| BDEST01P1_Contig1203 | 2 | 2 | Putative NTGP4 |
| BDEST01P1_Contig1223 | 4 | 3 | Endo-1,4-beta-glucanase Cel1 |
| BDEST01P1_Contig1229 | 2 | 7 | Putative phospho-2-dehydro-3-deoxyheptonate aldolase 1,chloroplast precurser |
| BDEST01P1_Contig1272 | 7 | 8 | Coated vesicle membrane protein-like |
| BDEST01P1_Contig1275 | 4 | 6 | Hypothetical protein |
| BDEST01P1_Contig1335 | 2 | 4 | Putative uncharacterized protein Os04g0692000 protein |
| BDEST01P1_Contig1400 | 5,2,7 | 9 | Heat shock protein 80 |
| BDEST01P1_Contig1408 | 7 | 7 | Thiamine biosynthetic enzyme |
| BDEST01P1_Contig1423 | 6 | 2 | Receptor protein kinase PERK1-like protein |
| BDEST01P1_Contig1458 | 5 | 9 | Putative poly(A)-binding protein |
| BDEST01P1_Contig1503 | 1 | 6 | Actin-7 |
| BDEST01P1_Contig1535 | 4 | 3 | 40S ribosomal protein S21 |
| BDEST01P1_Contig1567 | 4,1,5 | 7 | Alpha tubulin-1B |
| BDEST01P1_Contig1574 | 6 | 6 | Putative 60S ribosomal protein L13E |
| BDEST01P1_Contig1612 | 7,3 | 5 | Putative uncharacterized protein |
| BDEST01P1_Contig1738 | 4 | 3 | RNA recognition motif family protein, expressed |
| BDEST01P1_Contig1760 | 3 | 1 | Hypothetical protein |
| BDEST01P1_Contig1847 | 3 | 1 | Hypothetical protein |
| BDEST01P1_Contig1853 | 6,1,5 | 1 | Unknown |
| BDEST01P1_Contig1864 | 4 | 3 | Cell division cycle protein 48, putative, expressed |
| BDEST01P1_Contig1867 | 3 | 1 | Hypothetical protein |
| BDEST01P1_Contig1889 | 4 | 3 | Roothairless 3 |
| BDEST01P1_Contig1900 | 5 | 7 | Hypothetical protein |
| BDEST01P1_Contig1917 | 2 | 2 | Ribosomal subunit 8E protein |
| BDEST01P1_Contig1922 | 5 | 3 | Lipoxygenase 1 protein |
| BDEST01P1_Contig1933 | 5,2 | 2 | Hypothetical protein |
| BDEST01P1_Contig1936 | 3,1,4 | 10 | Hypothetical protein |
| BDEST01P1_Contig1938 | 1 | 12 | Eukaryotic translation initiation factor 2 gamma subunit, putative, expressed |
| BDEST01P1_Contig1943 | 1 | 5 | Unknown protein |
| BDEST01P1_Contig1960 | 5,7 | 11 | CBL-interacting serine/threonine-protein kinase 15, putative, expressed |
| BDEST01P1_Contig1991 | 4,5 | 3 | Ubiquitin family protein, expressed |
| BDEST01P1_Contig2089 | 2,1 | 7 | Hypothetical protein |
| BDEST01P1_Contig2103 | 4,3 | 3 | Tonoplast intrinsic protein1 |
| BDEST01P1_Contig2128 | 1 | 1 | Putative 26S proteasome subunit RPN9b |
| BDEST01P1_Contig2133 | 6 | 2 | Ribosomal protein L6 |
| BDEST01P1_Contig2194 | 5,4 | 3 | Aconitate hydratase, cytoplasmic putative, expressed |
| BDEST01P1_Contig2253 | 4 | 3 | Hypothetical protein |
| BDEST01P1_Contig2261 | 4,2 | 3 | Hypothetical protein |
| BDEST01P1_Contig2263 | 1 | 1 | Hypothetical protein |
| BDEST01P1_Contig2264 | 2 | 7 | Expressed protein |
| BDEST01P1_Contig2288 | 2 | 3 | Hypothetical protein |
| BDEST01P1_Contig2298 | 1 | 10 | Ribosomal Pr 117 |
| BDEST01P1_Contig2330 | 5 | 3 | HTH DNA-binding protein |
| BDEST01P1_Contig2403 | 4 | 11 | Putative RING-H2 zinc finger protein |
| BDEST01P1_Contig2416 | 7 | 8 | Succinate dehydrogenase |
| BDEST01P1_Contig2475 | 7 | 9 | Putative monodehydroascorbate reductase |
| BDEST01P1_Contig2483 | 1 | 5 | Target of rapamycin |
| BDEST01P1_Contig2544 | 4,2 | 7 | Thioredoxin family-like protein |
| BDEST01P1_Contig2547 | 4,5,6 | 3 | Catalase-1, putative, expressed |
| BDEST01P1_Contig2610 | 6 | 2 | Translational elongation factor Tu |
| BDEST01P1_Contig2648 | 4,5 | 3 | Putative uncharacterized protein |
| BDEST01P1_Contig2690 | 4 | 3 | Mitochondrial processing peptidase beta subunit |
| BDEST01P1_Contig2693 | 2 | 4 | OSIGBa0096P03.7 protein |
| BDEST01P1_Contig2731 | 3 | 1 | Aspartate aminotransferase, cytoplasmic |
| BDEST01P1_Contig2741 | 5 | 9 | Putative glucose-6-phosphate isomerase |
| BDEST01P1_Contig2745 | 3 | 1 | Malate dehydrogenase |
| BDEST01P1_Contig2780 | 6 | 2 | Putative uncharacterized protein |
| BDEST01P1_Contig2795 | 2 | 7 | Calcium-dependent protein kinase |
| BDEST01P1_Contig2836 | 1,5,6 | 1 | Unknown |
| BDEST01P1_Contig2842 | 6 | 4 | Putative uncharacterized protein |
| BDEST01P1_Contig2844 | 4 | 3 | 50S ribosomal protein L15, chloroplast precursor (CL15) |
| BDEST01P1_Contig2870 | 2,4 | 3 | Pyruvate decarboxylase isozyme 2 |
| BDEST01P1_Contig2933 | 4 | 3 | EIF-2-beta |
| BDEST01P1_Contig2979 | 4,5 | 7 | Calmodulin |
| BDEST01P1_Contig2982 | 3,7 | 6 | Vacuolar proton-ATPase |
| BDEST01P1_Contig2993 | 5,6 | 9 | 1-aminocyclopropane-1-carboxylate oxidase |
| BDEST01P1_Contig3007 | 5 | 9 | Putative uncharacterized protein |
| BDEST01P1_Contig3009 | 1,3 | 1 | Guanine nucleotide-binding protein subunit beta-like protein |
| BDEST01P1_Contig3011 | 2 | 7 | Putative Serine/threonine Kinase |
| BDEST01P1_Contig3022 | 3 | 1 | Putative uncharacterized protein |
| BDEST01P1_Contig3037 | 6 | 8 | Putative uncharacterized protein |
| BDEST01P1_Contig3042 | 4 | 3 | Putative uncharacterized protein |
| BDEST01P1_Contig3062 | 3 | 1 | Putative selenium binding protein |
| BDEST01P1_Contig3090 | 2 | 11 | Serine hydroxymethyltransferase |
| BDEST01P1_Contig3139 | 4 | 3 | Putative ribosomal protein |
| BDEST01P1_Contig3155 | 3 | 1 | ATP synthase subunit beta |
| BDEST01P1_Contig3176 | 4 | 11 | Alpha tubulin |
| BDEST01P1_Contig3177 | 1,6 | 2 | Putative NEC1 |
| BDEST01P1_Contig3188 | 2 | 5 | Manganese superoxide dismutase |
| BDEST01P1_Contig3204 | 6 | 2 | Putative calcium-dependent protein kinase |
| BDEST01P1_Contig3229 | 2 | 2 | Ribosomal subunit 8E protein |
| BDEST01P1_Contig3230 | 6,7 | 6 | 26S proteasome ATPase subunit Rpt6 |
| BDEST01P1_Contig3231 | 4 | 3 | 30S ribosomal protein S1 |
| BDEST01P1_Contig3244 | 2 | 7 | Putative monosaccharide transport protein MST1 |
| BDEST01P1_Contig3247 | 5 | 7 | Putative uncharacterized protein |
| BDEST01P1_Contig3321 | 4 | 11 | Protein disulfide isomerase 2 precursor |
| BDEST01P1_Contig3332 | 2 | 3 | Sucrose synthase |
| BDEST01P1_Contig3337 | 4 | 3 | Actin-1 |
| BDEST01P1_Contig3341 | 3 | 1 | Phospholipase D alpha 1 precursor |
| BDEST01P1_Contig3349 | 3,4,7 | 2 | Putative elongation factor 2 |
| BDEST01P1_Contig3368 | 5 | 3 | Alpha-tubulin 4 |
| BDEST01P1_Contig3396 | 4,7 | 6 | Putative uncharacterized protein |
| BDEST01P1_Contig3409 | 5 | 3 | J-domain protein |
| BDEST01P1_Contig3419 | 3 | 11 | 14-3-3E |
| BDEST01P1_Contig3425 | 6,7 | 6 | Eukaryotic initiation factor 4A |
| BDEST01P1_Contig3448 | 7 | 6 | Filamentation temperature-sensitive H 2B |
| BDEST01P1_Contig3524 | 3 | 1 | Tubulin beta-2 chain |
| BDEST01P1_Contig3535 | 5 | 9 | Aldehyde dehydrogenase |
| BDEST01P1_Contig3596 | 7 | 3 | Translation elongation factor-1 alpha |
| BDEST01P1_Contig3628 | 5 | 2 | TPA: putative cysteine proteinase precursor |
| BDEST01P1_Contig3670 | 3 | 4 | 14-3-3-like protein GF14-B |
| BDEST01P1_Contig3684 | 7 | 3 | EF-1 alpha |
| BDEST01P1_Contig3706 | 7 | 8 | Putative aminotransferase |
| BDEST01P1_Contig3714 | 4,5 | 12 | UDP-glucose 6-dehydrogenase |
| BDEST01P1_Contig3715 | 5 | 12 | Ribosomal protein L3 |
| BDEST01P1_Contig3720 | 7 | 6 | Catalase isozyme 1 |
| BDEST01P1_Contig3721 | 3,4,7 | 6 | Cytosolic 6-phosphogluconate dehydrogenase |
| BDEST01P1_Contig3724 | 1,3,5 | 11 | Actin |
| BDEST01P1_Contig3747 | 6 | 2 | Phenylalanine ammonia-lyase |
| BDEST01P1_Contig3763 | 6 | 2 | ATP/ADP translocator |
| BDEST01P1_Contig3786 | 1,4,5 | 3 | Alpha tubulin |
| BDEST01P1_Contig3798 | 7 | 2 | Hypothetical protein |
| BDEST01P1_Contig3812 | 2 | 6 | Polyubiquitin |
